# Co-use of Alcohol and Tobacco Among Ninth-Graders in Louisiana

**Published:** 2009-06-15

**Authors:** Carolyn C. Johnson, Larry S. Webber, Leann Myers, Neil W. Boris, Gerald S. Berenson

**Affiliations:** Tulane School of Public Health and Tropical Medicine, Community Health Sciences; Tulane University School of Public Health and Tropical Medicine, New Orleans, Louisiana; Tulane University School of Public Health and Tropical Medicine, New Orleans, Louisiana; Tulane University School of Public Health and Tropical Medicine, New Orleans, Louisiana; Tulane University School of Public Health and Tropical Medicine, New Orleans, Louisiana

## Abstract

**Introduction:**

The co-use of alcohol and tobacco by adolescents is a public health problem that continues well into adulthood and results in negative behavioral, social, and health consequences. The purpose of this study was to examine the co-use of alcohol and tobacco among ninth-graders in south-central Louisiana.

**Methods:**

We created a health habits survey to collect data from 4,750 ninth-grade students, mean age 15.4 years. Cross-sectional analysis used *χ* ^2^, 1-way analysis of variance, and logistic regression methods.

**Results:**

Almost 20% of students were co-users. Students who were white, performed poorly in school, did not expect to graduate high school, and had more discretionary money to spend were more likely to be co-users. Co-users had friends who got drunk weekly and were more likely to approve of alcohol use among friends than among adults. Significant differences in attitudes toward drinking and smoking were observed between co-users and nonusers. For adolescent drinkers, including girls, hard liquor was the preferred beverage.

**Conclusion:**

These data for high school students are applicable for prevention strategies at a critical age when harmful health behaviors can mark the start of lifelong habits. Intervention efforts will be successful only if they account for multiple levels of influence.

## Introduction

Adolescent alcohol and tobacco use are public health problems. Federal legislation in 1984 mandated 21 years as the legal age for purchasing and drinking alcohol, and the legal age for purchasing tobacco is 18 years in most states (1-3). Numerous studies have shown, however, that adolescents who are younger than 18 have easy access to both alcohol and tobacco, and availability is so widespread among youth that targeting availability may be a failed intervention strategy (4-6). Longitudinal data show that the prevalence of alcohol and tobacco use among adolescents escalates into adulthood and does not begin to decrease until older ages (7).

In 2004, the US Surgeon General stressed that tobacco use causes diseases in nearly every organ of the body (8), and alcohol use in adolescence can lead to violence (including homicides and suicides), drowning, sexually transmitted diseases, school failure, and motor vehicle crashes. Driving while under the influence of alcohol is the leading cause of death in the United States for people aged 16 to 24 years (9,10). Extensive research has examined adolescent drinking and smoking separately, but data regarding the co-use of alcohol and tobacco are sparse (7).

Researchers have addressed psychosocial risk factors, patterns of initiation and maintenance, and even physiological and genetic contributions (11-15) for the use of tobacco and alcohol separately. Characteristics of adolescents who use alcohol and tobacco concomitantly need to be identified because these unhealthy behaviors often occur together.

We conducted a tobacco use prevention program, Acadiana Coalition of Teens Against Tobacco (ACTT), for high school students in south-central Louisiana. Participation by youth in national surveys has traditionally been low in this area (16,17). In this study we examined the concomitant use of alcohol and tobacco among adolescents in Acadiana during their first year of high school.

## Methods

### Design

We defined the ACTT cohort as all students enrolled in ninth grade in 24 public schools in 6 school districts and conducted baseline measurement on 4,763 students, average age 15 years. Twenty-two of the 24 schools contacted agreed to participate. We used 2 schools to develop and test instruments and intervention activities. After baseline measurement, we randomized participating schools to intervention or control conditions; therefore, these data are not reported by condition but in the aggregate.

### Health Habits Survey

The Health Habits Survey was developed by a committee consisting of the principal investigator, co-investigators, and program staff. Items were extracted from Monitoring the Future (17), the Youth Risk Behavior Surveillance System (18), and the Bogalusa Heart Study (19). The final document (5) contained a total of 54 items in 5 sections: demographics, tobacco and alcohol use, attitudes/beliefs about smoking and drinking, and social relationships involving alcohol and tobacco (Appendix). Demographic items were age, sex, race, academic performance (students were asked if their grades were mostly A's, B's, C's, D's, or F's), immediate goals (*Looking ahead, what are your most immediate goals?*), discretionary money (*How much money do you usually spend per week any way you want?*), and physical activity (the question was *On how many of the past 7 days did you exercise or participate in physical activities for at least 20 minutes that made you sweat and breathe hard?*). The following questions assessed 30-day prevalence for alcohol and tobacco use: *During the past 30 days, how often have you had at least 1 beer, 1 glass of wine, or 1 shot of liquor?* and *During the past 30 days, have you smoked at least 1 cigarette?* For questions regarding attitudes and beliefs about alcohol and tobacco use, Likert scale responses were obtained (Appendix).

With approval from the Tulane University institutional review board, students participated in the Health Habits Survey with "passive" consent; that is, parents signed and returned the form only if they objected to participation. Students also completed an assent form at time of administration. Staff trained in standardized protocols administered surveys in classrooms or assemblies, and a second administration was scheduled if participation was initially low. Using school enrollment lists, we randomly assigned each student a unique 5-digit identification number before survey administration.

### Statistical analyses

We used *χ* ^2^ analysis to evaluate prevalence, race, sex differences, and categorical outcomes, and 1-way analysis of variance for continuous outcomes. When the distributional assumptions of analysis of variance were not met, the Kruskal-Wallis test was used. We used logistic regression methods to model alcohol prevalence as a function of relationship variables. All analyses were performed with SAS version 9.1 (SAS Institute, Inc, Cary, North Carolina). We included all students in the calculation of demographic frequencies, but those who self-reported as Latino, Asian American, American Indian, or "other" were excluded from additional analyses because of small sample sizes.

## Results

### Sample

Of 5,156 available ninth-graders, 4,808 responded to the survey, for a participation rate of 93.3%. Nonresponse was due to parental or student refusals (or both) and student absences during the survey administration. Another 58 students' data were deleted because of missing sex, race, or alcohol or tobacco use data, resulting in 4,750 students for analysis.

Students' mean age was 15.4 years (range, 11.8-19.3 years), and 51% were female. The sample was mainly white (61.1%) and African American (32.8%), a distribution consistent with many populations throughout the southeastern United States (20). Slightly more than 1% each was Latino, Asian, and Native American, and 1.9% reported "other." The sex distribution was similar in all ethnic groups except for Native Americans. The prevalence of ever having used alcohol was 37.5% among nonsmokers but was 79.5% among smokers (*P* < .001), indicating that the 2 behaviors clustered much higher than would be expected by chance. Approximately 34% of the total sample of adolescents who reported alcohol use preferred liquor, followed by wine (25.1%), then beer (18.2%). The number of alcoholic drinks consumed was related to the frequency of alcohol consumption (not shown). More than half of the cohort (52%) reported they did not disapprove of their friends taking 1 or 2 drinks every day, but 57% disapproved of adults having 1 or 2 drinks every day (not shown).

### Co-use of alcohol and tobacco

Of the African American and white students combined (n = 4,431), 879 (19.8%) reported being co-users. We examined sociodemographic characteristics for 4 tobacco and alcohol use categories: co-users (n = 879), nonusers (n = 2,079), smokers/nondrinkers (n = 227), and drinkers/nonsmokers (n = 1,246). Alcohol use was defined as drinking any alcoholic beverage in the last 30 days, and tobacco use was defined as having smoked at least 1 cigarette in the last 30 days. Comparisons of nonusers and co-users (Table 1) showed no sex difference in any of the 4 categories, but whites (26%) were more likely than African Americans (8.4%) to be co-users (*P* < .001). Co-users were more likely to report earning mostly D's and F's for school grades (*P* < .001), and they were less likely to report expecting to graduate from high school. Co-users also had more discretionary money to spend (*P* < .001). Students who smoked but did not drink reported the fewest physically active days in the past week (*P* < .002); however, no significant differences in physically active days were observed for co-users compared with the other groups.

We analyzed social relationships for all of the smoking/drinking categories, but only the comparisons between nonusers and co-users are shown (Table 2). (For the actual questions that contribute to the derived values, see Table 2 footnotes and the Appendix.) Approximately 81% of co-users indicated that most or all of their friends were likely to drink alcohol, compared with 27.9% of nonusers. Co-users reported having more friends who drink alcohol (*P* < .001) and even having more friends who drink weekly and who smoke or chew tobacco (*P* < .001). Two-thirds of co-users reported that most or all of their friends smoked.

Almost all co-users (91.3%) were either often or a few times with people who "drink for kicks," and about half of nonusers (52.3%) reported the same. Two-thirds of co-users had parents who smoked. Siblings of co-users were more likely to smoke and chew tobacco compared to siblings of nonusers and the other 2 groups. Comparisons between all of the smoking/drinking groups (including nondrinkers/smokers and nonsmokers/drinkers) showed similar patterns; percentages of nondrinkers/smokers were higher than drinkers/nonsmokers and nonusers, but lower than co-users (not shown). The only frequency comparisons that were not significantly different among the nondrinker/smoker and nonsmoker/drinker groups were those for chewing tobacco by parents, siblings, or friends.

We compared attitudes and beliefs about alcohol and tobacco use of co-users with those of the other 3 categories of use/nonuse (Table 3 compares nonusers with co-users). We found significant differences between co-users and nonusers. For alcohol and tobacco disapproval, risk of harm, and what friends think, higher scores reflected stronger disapproval. For these attitudes, scores were consistently highest for nonusers and consistently lowest for co-users. Nonusers had the highest level of disagreement with the statements that smoking will hurt you only if you inhale and smoking will not hurt you if you do not smoke too much. Co-users had the lowest levels of disagreement with these statements. Comparisons with the other 2 groups (drinker/nonsmoker and smoker/nondrinker) reflected the same general pattern at the *P* = .001 level; means for drinkers/nonsmokers were closest to those for the nonuser category and means for nondrinkers/smokers were closest to those for the co-user category (not shown). The only comparison not significant was that between co-users and nondrinkers/smokers, where means were similar.

## Discussion

Demographic, social, and attitudinal profiles developed in this study identify adolescents who are at high risk for alcohol and tobacco use. This information can help improve intervention strategies for adolescents (21). Alcohol and tobacco may act as gateway agents, individually or together, to further drug use (21).

The adolescent preference for hard liquor over beer or wine is a matter of concern. In the 1990s, beer was the adolescent drink of choice (19,22). Brewers such as Anheuser-Busch now view the recent shift toward hard liquor as a business challenge (23). Drinking hard liquor is a problem for adolescents because a 1- to 2-ounce shot, even with 6 ounces of a mixer, can deliver more ethanol to the system than a 12-ounce beer or 6-ounce glass of wine (19). The trend toward hard liquor is perplexing because hard liquor cannot be advertised on network television. Some cable channels, however, such as Spike TV, do advertise hard liquor. Drink preferences have changed and differences between male and female drinking have narrowed, raising the question of associations between the 2 trends. For example, a study in England found that preference for "spirits" increased with age and was higher among girls than boys (24).

Ninth-grade students were less likely to disapprove of their friends drinking 1 or 2 drinks every day than they were to disapprove of adults doing the same. This finding is intriguing and could imply that adolescents are more concerned about parental drinking than they are about their own or their friends' drinking.

In the Cajun culture in the Acadiana area of Louisiana, smoking and drinking alcohol are common among adults as well as young people. Therefore, the cultural anomaly is that these behaviors can be considered normal rather than risky (25). The role of sociocultural factors in adolescent smoking and drinking is widely recognized (13), and it is not surprising that as many as one-fifth of the high school students in this region of Louisiana reported co-use of alcohol and tobacco.

Both girls and boys are co-users. These data are startling because for many years, more boys than girls drank and smoked. Smoking data have been more variable (17,24); smoking rates for girls have been increasing at high school age. No difference between male and female smoking in the junior and senior high school years has been observed in some regional and local studies (26), but others report higher smoking rates in girls (24). Amos and Bostock (2007) compared reasons boys and girls smoke to determine why girls' smoking rates are increasing; they found that girls smoke to alleviate stress and to maintain or lose weight, and girls consider smoking an integral part of socializing and "social sharing" (27).

More white than African American adolescents were co-users. This finding is not surprising and is consistent with national and local data (5,16,17,27). Co-users were more likely to earn D's and F's in school and less likely to intend to graduate from high school. They also had the most discretionary money to spend.

Not surprising are the data showing that more co-users have friends who drink and who drink "for kicks." What is disturbing is the frequency with which those friends reportedly get drunk (ie, weekly). Co-users also have more friends who smoke cigarettes, chew tobacco, or both. Consistent with the smoking literature (19), family members of co-users, parents as well as siblings, were reported to be more likely to smoke cigarettes or chew tobacco. Several studies have shown that the most consistent risk factors for initiating drinking and smoking in adolescence are parental approval and models for drinking and drug use (28). Some studies, though, show that these social models are more influential early in life or at time of initiation rather than over the long term (29,30). It has recently been argued that genetic factors, which account for parental influence and drive selection of risk-taking friends, may be more important than social modeling for influencing adolescent smoking and drinking (31). Once use of nicotine and alcohol has started, shared behavioral and neurobiological factors may enhance cross-dependence (12).

Obviously, the co-user is less likely to disapprove of smoking and drinking or of anyone else who smokes and drinks. The co-user is also more likely to agree that smoking is harmful only if you smoke too much or inhale but less likely to agree that buying cigarettes is a waste of money or disgusting. The means for reported variables of interest were similar for smokers across the 2 categories in which smoking was involved.

Our data present a picture of the adolescent co-user as having social relationships and family members that reinforce and support smoking and drinking behaviors and attitudes. Any intervention effort that targets the co-user will have difficulty achieving positive results unless the intervention occurs at multiple levels, such as personal, family, friends, and cultural environment. Neither the Youth Risk Behavior Surveillance System nor Monitoring the Future report data on co-use of alcohol and tobacco; therefore, the value of this study is to provide information for use by schools and health educators in developing education and prevention programs and for challenging the cultural norms that place students at high behavioral risk. Because limited data are available in this area of Louisiana, the study of adolescents in Acadiana provides valuable insights for curbing smoking and drinking as a serious health and behavioral risk for future cardiovascular disease, malignancies, and self-defeating behaviors.

The reported data are cross-sectional; therefore, data are discussed as associative, not causative. Traditionally, the Acadians in south-central Louisiana have been a difficult-to-reach population, and for this reason, these data are especially valuable. On the other hand, the culture is somewhat unusual, especially in the early ages at which adolescents engage in adult risk behaviors; therefore, the study results may not be generalizable to adolescent populations in other areas of the United States.

## Figures and Tables

**Table 1:** Demographic Characteristics by Smoking and Drinking Behaviors Among 4,431 White and African American Ninth-Graders, Acadiana Coalition of Teens Against Tobacco

**Characteristics**	**Nondrinkers and Nonsmokers (n = 2,079)**	**Drinkers and Smokers (n = 879)**	***P* Value^a^**
**Sex, n (%)**			
Male	989 (47.6)	438 (49.9)	.26
Female	1090 (52.4)	441 (50.1)	
**Ethnicity, n (%)**			
White	1179 (56.7)	750 (85.3)	<.001
African American	900 (43.3)	129 (14.7)	
**Grades, n (%)**			
A's	482 (25.9)	101 (12.8)	<.001
B's	658 (35.4)	222 (28.1)	
C's	622 (33.5)	361 (45.6)	
D's-F's	95 (5.1)	107 (13.5)	
**Goals, n (%)**			
Drop out	31 (1.5)	44 (5.1)	<.001
Graduate	1978 (96.2)	773 (89.9)	
Other	47 (2.3)	43 (5.0)	
**Money, $ mean (SD)^b^**	12.70 (9.5)	18.10 (9.0)	<.001
**Days active past week, mean (SD)**	3.6 (2.1)	3.6 (2.2)	.93

Abbrevations: NS, not significant.

a *Χ* ^2^ test for frequencies, Kruskal-Wallis for continuous data, Bonferroni adjusted.

b "How much money do you usually spend per week any way you want?"

**Table 2:** Social Relationships by Smoking and Drinking Behaviors Among 4,431 White and African American Ninth-Graders, Acadiana Coalition of Teens Against Tobacco

**Characteristics**	Nondrinkers and Nonsmokers (n = 2,079) n (%)	**Drinkers and Smokers (n = 879) **n (%)	*P* Value^a^
**Parents smoke**			
No	1215 (58.4)	293 (33.3)	<.001
Yes	864 (41.6)	586 (66.7)	
**Siblings smoke**			
No	1813 (87.2)	510 (58.0)	<.001
Yes	266 (12.8)	369 (42.0)	
**Friends smoke**			
None/some	1844 (89.3)	295 (33.9)	<.001
Most/all	221 (10.7)	674 (66.1)	
**Friends drink**			
None/some	1488 (72.1)	162 (18.6)	<.001
Most/all	576 (57.9)	707 (81.4)	
**Parents chew**			
No	1901 (91.4)	759 (86.3)	<.001
Yes	178 (8.6)	120 (13.7)	
**Siblings chew**			
No	2017 (97.0)	802 (91.2)	<.001
Yes	62 (3.0)	77 (8.8)	
**Friends chew**			
None/some	2007 (97.2)	782 (90.2)	<.001
Most/all	57 (2.8)	85 (9.8)	
**Friends drunk^c^**			
None/some	1917 (93.0)	547 (62.9)	<.001
Most/all	144 (7.0)	323 (37.2)	
**For kicks^d^**			
Not at all	984 (47.7)	75 ( 8.7)	<.001
A few times/often	1078 (52.3)	792 (91.3)	

a *Χ* ^2^ test.

b Bonferroni adjusted.

c Question: "How many of your friends get drunk at least once a week?"

d Question: "During the past 12 months how often have you been around people who were using alcoholic beverages to get high or for 'kicks'?"

**Table 3:** Attitudes About and 30-Day Prevalence of Smoking and Drinking, Acadiana Coalition of Teens Against Tobacco

Variable With Component Questions	**Nondrinkers and Nonsmokers n = 2,052**	**Drinkers and Smokers n = 870**	*P *Value^a^

	Mean (SD)	Mean (SD)	
Tobacco disapproval^b^	6.9 (1.9)	5.0 (1.8)	<.001
Alcohol disapproval^b^	5.9 (2.0)	4.0 (1.4)	<.001
Risk of harm^b^	12.6 (2.7)	10.6 (2.9)	<.001
Friends^b^	9.1 (2.7)	6.5 (2.2)	<.001
Cigarettes are waste of money/Smoking is disgusting^c^	3.5 (2.1)	6.2 (2.1)	<.001
Smoking hurts only if smoke too much/only if inhale^c^	7.7 (2.0)	6.5 (1.8)	<.001

a Higher scores reflect stronger disapproval.

b Higher scores reflect stronger agreement with the two statements.

c Kruskal-Wallis test. Derived variables:"Tobacco disapproval" (combined 3 items questioning disapproval of people who smoke 1 or more packs of cigarettes per day, use smokeless tobacco regularly, and who are 18 or older smoking 1 or more packs of cigarettes per day; scale for all items 3 to 9). "Alcohol disapproval" (combined 3 items questioning disapproval of people who take 1 or 2 drinks of alcoholic beverages every day, who have 5 or more drinks every weekend, and who are 18 or older taking 1 or 2 alcoholic beverages nearly every day; scale for all items 3 to 9). "Harm" (combined 4 items questioning how much do you think people risk harming themselves if they smoke 1 or more packs of cigarettes a day, use smokeless tobacco regularly, take 1 or 2 drinks of alcoholic beverages every day, and have 5 or more drinks every weekend; scale for all items 4 to 16). "Friends" (combined 4 items questioning how you think your close friends feel about you if you smoke 1 or more packs of cigarettes a day, use smokeless tobacco regularly, take 1 or 2 drinks of alcoholic beverages every day, and have 5 or more drinks every weekend; scale for all items 3 to 12).

## Appendix

The Health Habits Survey for the Acadiana Coalition of Teens Against Tobacco Study is available for download as a Microsoft Word document. You must have Microsoft Word to use this file.

Download the Health Habits Survey (DOC 81k)
